# Qualitative Assessment of a Smartphone-Based Mobile Health Tool to Guide Diarrhea Management in Bangladesh

**DOI:** 10.4269/ajtmh.23-0444

**Published:** 2023-12-11

**Authors:** Debashish Biswas, Asadullah Asadullah, Sazzad Hossain Khan, Zahid Hasan Khan, Md Taufiqul Islam, Ashraful Islam Khan, Firdausi Qadri, Eric J. Nelson, Melissa H. Watt, Daniel T. Leung

**Affiliations:** ^1^ International Centre for Diarrhoeal Disease Research, Bangladesh (icddr,b), Dhaka, Bangladesh;; ^2^ Departments of Pediatrics and Environmental and Global Health, Emerging Pathogens Institute, University of Florida, Gainesville, Florida;; ^3^ Department of Population Health Sciences, University of Utah, Salt Lake City, Utah;; ^4^ Division of Infectious Diseases, University of Utah School of Medicine, Salt Lake City, Utah

## Abstract

Diarrheal diseases are a major cause of morbidity and mortality in children worldwide and a significant contributor to antimicrobial resistance. In the absence of laboratory diagnostics to establish diarrhea etiology, electronic clinical decision support tools can help physicians make informed treatment decisions for children with diarrhea. In Bangladesh, we assessed the feasibility and acceptability of an electronic Diarrhea Etiology Prediction algorithm (DEP tool) embedded into a rehydration calculator, which was designed to help physicians manage children with diarrhea, including decisions on antibiotic use. A team of Bangladeshi anthropologists conducted in-depth interviews with physicians (*N* = 13) in three public hospitals in Bangladesh about their experience using the tool in the context of a pilot trial. Physicians expressed positive opinions of the DEP tool. Participants perceived the tool to be simple and easy to use, with structured guidance on collecting and entering clinical data from patients. Significant strengths of the tool were as follows: standardization of protocol, efficiency of clinical decision-making, and improved clinical practice. Participants also noted barriers that might restrict the widespread impact of the tool, including physicians’ reluctance to use an electronic tool for clinical decision-making, increasing work in overburdened healthcare settings, unavailability of a smartphone, and patients’ preferences for antibiotics. We conclude that an electronic clinical decision support tool is a promising method for improving diarrheal management and antibiotic stewardship. Future directions include developing and implementing such a tool for informal healthcare physicians in low-resource settings, where families may first seek care for pediatric diarrhea.

## INTRODUCTION

Diarrheal diseases are a leading cause of morbidity and mortality among children in low- and middle-income countries (LMICs).^1^ Although the number of diarrheal deaths globally has decreased over recent years, appropriate and prompt treatment of diarrheal diseases remains a priority. According to the WHO’s guidelines for the management of diarrheal disease, antibiotics may be needed, but to mitigate antibiotic resistance, their use should be restricted to cases of bloody diarrhea or suspected cholera.^2^

The clinical decision-making process to treat a diarrheal illness with an antibiotic is often empiric. In LMICs, etiological diagnoses are rarely made, due to the prohibitive costs of laboratory diagnostics, and individual signs traditionally associated with bacterial causes, such as bloody diarrhea, may not be sufficiently sensitive to detect all cases of bacterial diarrhea. For example, in the multicenter Global Enteric Multicenter Study, the detection of *Shigella* ranged from 16% to 78% among children with dysentery (bloody diarrhea) and 2% to 43% among children with nonbloody diarrhea.^3^ This uncertainty around discerning which patients may benefit from antibiotics contributes to a large number (up to 70%) of patients with diarrhea being prescribed antibiotics.^4^ Inappropriate use of antibiotics leads to unnecessary toxicity for the individual, increased costs, and the risk of antibiotic resistance in the community.^5^ Data from the Global Burden of Disease study estimates that almost 5 million deaths were associated with bacterial antimicrobial resistance in 2019, making antimicrobial stewardship a top priority for global health.^6^

Given the lack of clear clinical predictors to differentiate viral from nonviral agents, decisions for antibiotic use are typically based on several “rules of thumb” for which evidence is scant. Unfortunately, this gestalt poorly predicts the indication for antibiotics. A recent study of children presenting to Kenyan hospitals with diarrhea showed that reliance on dysentery as a proxy for *Shigella* infection led to the failure to diagnose shigellosis in nearly 90% of cases.^7^

Better tools for decision-making and evidence-based guidelines regarding antibiotic use in children with diarrhea are clearly needed. Mobile health (mHealth) tools can fill this gap by bringing clinical algorithms to the bedside to guide decision making. The goal of this study was to qualitatively examine the acceptability and feasibility of a clinical decision support tool to help physicians appropriately treat children with diarrhea in Bangladeshi hospitals, including decisions about the use of antibiotics.

## MATERIALS AND METHODS

### Overview.

The study team, consisting on investigators from International Center for Diarrheal Disease Research, Bangladesh (icddr,b); Institute of Epidemiology, Disease Control and Research, University of Utah; and University of Florida, conducted a randomized crossover trial in public hospitals in Bangladesh to examine the effectiveness of the Diarrheal Etiology Prediction (DEP) tool, a smartphone-based tool used to manage pediatric diarrhea.^8^ The DEP tool combines a DEP algorithm for calculating the probability of a diarrheal case of being of viral etiology with a rehydration calculator to inform rehydration treatment. The DEP tool was designed with the intention of helping physicians make appropriate treatment decisions, including antibiotic use, for young children presenting with a primary complaint of diarrhea.^9^ In this study, we were interested in exploring the perceptions of physicians who used the DEP tool in the hospital setting. We conducted qualitative in-depth interviews (IDIs) at two time points, pre- and post-DEP tool use and implementation, to explore physicians’ experiences of the tool, with a focus on acceptability and feasibility.

### Setting.

The study was conducted at three government hospitals in Bangladesh. The study sites included 1) a district-level hospital in Narayanganj with approximately 100 inpatient beds, 2) a district-level hospital in Tangail with approximately 250 inpatient beds, and 3) a specialized tertiary hospital for infectious diseases in Chattogram with approximately 120 inpatient beds. The study sites were selected from the existing nationwide cholera surveillance sites in Bangladesh.^10^ All sites had previously participated in an mHealth-related study that deployed only the rehydration calculator portion of the tool and did not include diarrheal disease management without the DEP algorithm.^11^

### The DEP tool.

The DEP tool is a mobile phone–based application that provides physicians with the probability that a case of pediatric diarrhea is caused by a viral agent. The tool provides a prediction based on individual clinical variables (age, presence of bloody diarrhea, breastfeeding, vomiting, mid–upper arm circumference) and location-specific factors related to weather and seasonality. The DEP tool was incorporated into a rehydration calculator that was previously evaluated in two clinical studies in Bangladesh.^8^ The calculator provided recommendations for rehydration based on age, sex, weight, dehydration status, and previously provided fluids. For this study, the DEP tool did not provide antibiotic recommendations, only the probability that the case was caused by a virus.

The DEP tool was evaluated using a randomized crossover study, where physicians were randomized to periods where they used the DEP tool or a rehydration calculator application that did not include the DEP algorithm.^8^ The crossover included a washout period to reduce carryover effect. The study was conducted over a 9-weeks period.

### Study population.

We recruited participants from the pool of clinicians who had the responsibility of treating children with diarrhea at each site. Providers were identified in collaboration with the hospital leadership, with a goal to recruit the providers who are most likely to be seen for emergency/outpatient cases of pediatric diarrhea.

### Qualitative data collection.

A team of Bangladeshi anthropologists conducted in-depth interviews with participants to explore their perspectives on the feasibility of the DEP tool in hospital settings, as well as challenges and opportunities to improve diarrheal management in the public hospitals of Bangladesh. Participants were enrolled at the end of the first phase of the crossover study and were invited to participate in an additional interview at the end of the second phase.

The team conducted interviews at times that were convenient for study participants, being mindful to avoid disrupting their clinical workflow. The semistructured interview guide explored participants’ perceptions, experience, and thoughts related to the DEP tool they used over a period of 9 weeks and how this influenced their decision-making related to diarrheal management.

## DATA ANALYSIS

To identify and describe the themes that emerged in the interviews, we used thematic content analysis, an inductive analytic approach.^12^ Analysis was conducted iteratively throughout the data collection period to assess for thematic saturation. Most interviews (*N* = 10) were audio-recorded; in seven cases, the participants declined audio-recording. The study anthropologists took detailed handwritten notes, regardless of audio status, and expanded the notes after the interview. For audio-recorded interviews, the interviewer reviewed the recordings and wrote detailed notes summarizing the emerging themes, with retaining direct quotations to capture the full context and meaning of the discussion. All interviews were conducted in Bengali, and summaries were directly transcribed into English. The primary author (D. B.) along with second (A. A.) and third (S. H. K.) authors read the summaries to identify inductive themes related to the research objective. These themes were then shared with the investigator team to review and come to a consensus. The primary author categorized all data as per these identified themes for interpretation.^13^

### Human subjects.

The study team sought the consent of hospital authorities to visit the hospitals and to recruit and interview physicians. The research staff explained the study to each of the study participants and obtained verbal informed consent. The study protocol was approved by the Ethical Review Committee of the icddr,b and the Institutional Review Board at the University of Utah.

## RESULTS

Among the 15 physicians enrolled in the crossover study, 13 of these physicians participated in IDIs. In the first phase, eight physicians (out of 13) had been exposed to the intervention (DEP tool with rehydration calculator) and five had been exposed to the comparison (rehydration calculator only). In the second phase, four participants were reinterviewed; of these, one had been exposed to the intervention, and three had been exposed to the comparison. Thus, a total of 17 interview sessions were conducted throughout the study period.

Nearly half of IDI participants were used as emergency medical officers. The remainder included assistant or associate professors, a consultant of medicine department, an assistant surgeon, and a residential medical officer (Table 1). The physician participants were responsible for providing services to patients either in emergency or inpatient/outpatient departments. Most participating physicians (11 of 13) were male.

**Table 1:** Number of interviews

Type of participants/physicians	No. of participants
Associate professor	1
Assistant professor	2
Assistant surgeon	1
Consultant	1
Emergency medical officer	6
Residential medical officer	2
Total	13

Overall, the enrolled physicians described use of the DEP tool as a positive experience. Most of the participants preferred using the DEP tool for diarrheal management compared with standard practice. All participants had experience using the tool during a patient consultation. Despite a largely positive response to the tool, some participants reported challenges with using the tool during patient consultations. Participants’ perspectives and experiences using the DEP tool are summarized here, including perceived value, feasibility for implementation, and recommendations for scale-up.

### Perceived value.

Three themes emerged in the participants’ perceived value of the DEP tool: 1) standardization of clinical protocols, 2) efficiency of clinical decision-making regarding antibiotic usage, and 3) improved clinical practice.

#### Standardization of protocol.

The most frequently stated advantage of the DEP tool was its ability to provide structured guidance to physicians on collecting clinical data from patients and doing a thorough dehydration assessment as per the WHO guidelines. Physicians felt this helped to correct gaps in their care management, as this participant described: “It is possible that one doctor can forget what to do next. In that time this application can remind him.”

In addition to standardizing clinical assessments, participants spoke about how the DEP tool facilitated the standardization of treatment. Most participants felt that the tool helped them to decide whether antibiotics were needed. Participants explained how physicians varied considerably in their treatment of diarrheal patients, specifically how physicians in district and subdistrict peripheral health centers are particularly nonadherent to the WHO guidelines for diarrhea treatment. Participants felt that widespread use of the DEP tool would standardize diarrheal disease treatment and management across health facilities. One participant explained: “Treatment depends on skills of individual and it is not expected that each of the physician would have the same skills in managing diarrhea. So, if everyone follows a specific guideline, then it can help to standardize the treatment in each of the facilities.”

#### Efficiency of clinical decision-making.

Participants explained that clinical decisions are typically made based on physical examinations, including the patient’s general condition and dehydration status. Common treatment practices for diarrhea patients included oral rehydration solution (ORS) and intravenous (IV) fluids in the case of perceived severe dehydration. Participants also added that patients with symptoms of severe dehydration and diarrhea including dry mouth, lips, and tongue; sunken eyes; fever; abdominal pain; nausea; and vomiting were sometimes given antibiotics. Many respondents (*n* = 8) reported that by using the DEP tool, they could more efficiently assess patients’ dehydration status and make a corresponding clinical decision about their management.

Participants described how they used the viral probability output on the DEP tool to assess the etiology of diarrhea. Participants consistently mentioned that the information on the tool was well organized and easy to use. One participant stated, “The tool is just a form and nothing new for us. All the issues added here are common for a physician managing diarrhea. I can easily put all the information into the app.”

Participants noted that the phone-based tool had the potential for even greater efficiency by task-shifting diarrheal disease management to lower cadres of staff, such as nurses. Because the tool provides consistent and clear result-specific indicators, it can support the involved of all staff in the management of patients with diarrhea. One participant explained: “In our public hospitals there is a lack of physicians, and particularly at night availability of a senior physician is less. However, sometimes patients with severe diarrhea visit at night and nurses called us on the phone and we provide treatment. In that case I think a senior nurse can use this application and provide primary treatment to the patients in case a senior physician is not available.”

#### Improved clinical practice.

The DEP tool, which indicates the probability that the etiology of diarrhea is viral, helped physicians to make evidence-based decisions about antibiotic use and to avoid unnecessary antibiotics. Some respondents reported that although they know when a patient should be provided with antibiotics according to the WHO guidelines, sometimes they provide antibiotics regardless to avoid secondary risks or infections or to meet the expectations of patient families. One participant explained: “My first experience with this tool was excellent. I was confused whether I will use antibiotic or not. But the tool was showing me the viral probability, so I treated the patient with the tool’s suggestion, and it worked very well.”

### Feasibility of implementation.

The themes that emerged for the feasibility of implementation represented both strengths, including ease of use, and potential weaknesses, including physicians’ reluctance to use a mobile phone tool, high burden of healthcare settings, lack of information technology (IT) infrastructure and other technology, and patients’ attitudes.

#### Ease of use.

All participants reported that the DEP tool was simple and easy to use. After using the tool, all the respondents mentioned that the functionality of the tool was straightforward in its ability to automate the assessment of dehydration status and diarrhea etiology. The tool allowed participants to easily collect data from patients, and participants felt that the tool could be used by any cadre/level of health worker, including nurses and sub-assistant community medical officers. For example, one participant explained that “I like the application because it is simple. It is like other mobile applications that we are using in our smartphone nowadays. I believe anyone can use this.”

#### Physicians’ reluctance.

Some participants noted that many physicians may be reluctant to use the DEP tool because they feel as though they have sufficient training and expertise in the management of diarrheal diseases and do not require the support of a clinical decision support tool. In these circumstances, participants expressed doubt that the tool would change the behavior of the physicians, including antibiotic usage practices. A participant explained: “I have 20 years of experience of clinical practice, and I know how to treat a diarrheal patient. This tool will not add new things for my clinical decision. However, it may be appropriate for people who have less training on the guideline and less experience in managing a diarrheal patient.”

Some participants noted that introducing the tool to physicians would require physicians to have motivation to learn how to operate the DEP tool. Participants who treated patients in the emergency department were less interested in learning how to operate the tool. Participants suggested that the DEP tool may not be best suited for the emergency department because of the large number of patients and diversity of health conditions but may be better suited for use in an inpatient setting.

#### High burden of healthcare settings.

Although the implementation of the DEP tool was largely accepted and used during the study period, all participants mentioned that routinely using a phone-based tool to manage patients in a busy hospital setting presents multiple challenges. They pointed to their numerous roles and responsibilities, including examining patients, relaying instructions to patients’ families, prescribing medication, and providing counseling. They expressed concern that the tool may take additional time, prevent them from seeing all patients, and hamper with their usual workflow. One participant explained that “In the morning shift there was overcrowding, and I have to see many patients within a short period of time. In that time, I cannot give much time to an individual. This application will take more time than I usually spend per patient.”

Participants shared that physicians in district-level emergency departments typically see more than 100 patients per day, representing a large variety of cases, including significant traumas. The environment is often chaotic, with patients vying for attention and physicians trying to be responsive to a variety of needs. Participants expressed concern that this busy and high-stress environment may not be the ideal setting for using the DEP tool, especially during the seasons when diarrheal diseases are common.

Participants also noted the challenges of limited human resources. They reported that patient management should involve a team, including a doctor for assessment and examination of the patient, prescription of medicine, and patient follow-up, but that much of the implementation should be done by nurses and other support staff. However, district- and subdistrict-level (*Upazila*) hospitals do not have adequate human resources to support doctors. Therefore, doctors often do not have the bandwidth to conduct the necessary physical examinations to follow the WHO guidelines. In that case, using the DEP tool might not be feasible in the absence of support staff assistance.

#### Lack of IT infrastructure and technology.

Some participants mentioned concerns about Internet access, which is sometimes unavailable or inconsistent in government hospitals. Additionally, it was noted that some physicians may not have an Android phone required to run the DEP tool. Ensuring adequate IT infrastructure to support the tool was seen as a costly barrier to implementation. For example, one participant explained, “Some doctors may not be interested to use their personal phone for their official duty. I see providing smartphone to all doctors by the government or hospital authority may help to overcome this and ensure use of the app.” However, this discussion revealed a need to clarify that the tool operates offline and online.

#### Patients’ attitude toward use of the application.

Patients’ attitudes toward the application emerged as both a barrier and facilitator. Some participants felt that patients may not have a problem with their information being entered into a digital platform and may appreciate the use of a digital tool in the development of their treatment strategy. However, participants were concerned that patients and/or their caretakers may not feel comfortable with the physician’s treatment decision being based on the DEP tool, especially if it precluded the use of antibiotics. Participants explained that patients often want a quick recovery and may not feel that treatment with ORS alone was adequate. Caregivers are understandably worried when their child has diarrhea, and they may get angry if a doctor provides patients with only ORS or oral medications rather than antibiotics. Many community members are aware of what medications are commonly used to treat diarrhea because these medications are easily accessible and readily available at drug shops. Caretakers will often ask for specific antibiotics, which can influence physicians’ treatment decisions at peripheral-level hospitals. To mitigate this, participants recommended increasing patient awareness of the potential harms of antibiotics.

#### Physician perceptions of caretaker opinions.

Respondents from Tangail and Narayangonj shared that their hospitals do not have a separate ward for pediatric diarrheal patients. They noted that a separate ward for diarrheal patients could make integrating the DEP tool into physician workflow more efficient, and that selectively using the tool for only some patients may cause nondiarrheal patients to think that they were being overlooked by the physician, including in the emergency department. For example, one participant explained that “if we use technology to treat [a] patient, [it] should be used for all patients. In the emergency department you know various type of patients come to take healthcare, and if they see we are using this application/technology for some of the children and not for other children then they may think that other children are getting better treatment.”

### Potential for use in the resource-poor settings.

Although participants largely agreed on the value of the tool, regarding the feasibility of implementing the tool in the clinical setting, several participants noted that the tool may be best deployed among community providers and dispensers of antibiotics. By the time children with diarrhea present to a formal healthcare facility, many had already received an antibiotic from a pharmacy or informal provider (such as a “village doctor”). When patients present to the hospital having already started an antibiotic, they often feel pressure to continue antibiotic use, regardless of the tool’s suggested treatment method. Thus, participants suggested that the DEP tool may be helpful for local pharmacists or village doctors who may not have the training or knowledge base for appropriate use of antibiotic or IV fluids. Participants felt that the DEP tool could be used to help these informal providers make appropriate treatment decisions, including for antibiotic use, in the absence of a medical doctor.

## DISCUSSION

In this study, physicians reflected on their experiences using the DEP tool, a clinical decision support tool that estimates the probability that an episode of diarrhea is caused by a virus, thus guiding antibiotic prescription practices. Physicians noted significant strengths of the tool, including standardization of protocol, efficiency of clinical decision-making, and improved clinical practice. Participants perceived the DEP tool to be simple and easy to use. However, they also noted barriers that might restrict the widespread impact of the tool, including physicians’ reluctance to use a phone-based tool, the high burden of healthcare settings, shortcomings of IT infrastructure and technology, and patients’ perceived attitudes of the tool.

The DEP tool received an overall positive response from physicians to support diarrheal management in Bangladeshi public hospitals. Physicians acknowledged the usefulness of the DEP tool to aid them in making decisions about clinical management and as a comprehensive way to provide structured guidance in gathering clinical data to determine the etiology of a diarrhea case.

Participants in the study generally supported the use of a phone-based tool to collect data and receive feedback. This is consistent with a study conducted in Tanzania that also supports the use of an electronic tool for supporting integrated management of childhood illness care, over a paper tool.^11^^,^^14^^,^^15^

Bangladesh has one of the lowest physician-to-patient ratios in the South Asian region, with an estimated 0.6 physicians per 1,000 patients.^16^^,^^17^ These human resource constraints provide both an opportunity and a limitation for the implementation of mHealth tools such as the DEP tool. On one hand, such tools can help in the task-sharing of diarrheal disease management to lower cadres of healthcare workers. There is evidence of mHealth applications facilitating task sharing. A recent randomized controlled trial found that a mobile application–assisted nurse-led model for asthma management improved clinical outcomes.^18^ However, there was concern that an mHealth tool would add to the workload of already stretched physicians by lengthening the patient encounter. To be effective and have sustained impact, it is necessary that an mHealth tool is embedded in and improves existing systems of care, without adding to providers’ burden.

There is evidence that receiving clinical decision support on diarrheal management can contribute to improved antibiotic stewardship. It was previously found that a smartphone-based tool on diarrheal disease management improved adherence to the WHO guidelines by decreasing IV fluid administration and improving rational use of antibiotics.^11^^,^^14^ Our data suggest that the rehydration calculator with the DEP tool has the potential to have increased impact because physicians expressed a willingness to adjust their clinical management based on the DEP tool.

However, the feasibility of the tool to have a broad impact on clinical practice may be restricted by some key challenges. In existing clinical practice (i.e., consultation, referral, and supervision) healthcare providers usually rely on presumptive treatment or an “educated guess” based on knowledge and expertise in the context of work experience.^19^^,^^20^ This often presents an obstacle to adopt an innovation in existing treatment practice. As a mobile phone–based application, the DEP tool requires a shift in practice. Physicians, particularly senior consultants, might feel reluctant to adopt this new application as a clinical decision support tool because it conflicts with normative practices for relating to patients and making clinical decisions. We found that emergency officers were particularly reluctant to use this tool because of concerns about delaying critical patient care. There is a need for further qualitative studies to identify other areas of potential intervention to improve antibiotic stewardship among physicians.

The current bed occupancy rate in Bangladesh is 0.8 per 1,000,^21^ with tertiary medical college hospitals having the highest and subdistrict (*Upazila*) health complexes having the lowest bed occupancy ratios. Moreover, more than 170 million outpatient visits occur each year in the country.^22^ Prior research in Bangladesh has identified overcrowding and human resource constraints as barriers to adherence to clinical guidelines.^23^ In our study, participants were concerned that the process of using the DEP tool, including the collection and input of required data, could compromise the ability to serve large patient loads.

Considering these challenges and opportunities, we recommend further research to determine the potential feasibility and acceptability of using the DEP tool among informal rural healthcare providers in the management of pediatric diarrhea. Informally trained healthcare providers provide up to 90% of all healthcare interactions in LMICs.^24^ In Bangladesh, informal providers represent up to 95% of healthcare providers nationally. Among such informal providers are village doctors, also known as rural medical practitioners, who practice mostly allopathic medicine without formal training.^25^ One study in a rural area of Bangladesh showed that village doctors were the first line of care for 65% of patients and the sole source of care for 46% of patients.^26^ Moreover, informal providers have high rates of antibiotic utilization for diarrheal illness in Bangladesh.

This study must be interpreted in the light of its limitations. First, the sample size was small, male participants were overrepresented, and follow-up was limited because of the COVID-19 pandemic. Therefore, the results may not be generalizable to other settings and situations. Implementation of the tool in other settings, with robust user feedback, may provide additional insights. Second, this study did not include patients’ perceptions, which may have offered a different and important perspective on the use of an mHealth tool in clinical practice. Physician participants speculated on the patient experience, but future studies should consider including patients’ voices. Third, social desirability bias was a strong possibility. All participants may have felt inclined to give positive feedback regarding the DEP tool.

Despite these limitations, our study demonstrates the potential for an mHealth approach such as the DEP tool to impact diarrheal management and antibiotic stewardship. The data suggest that this tool was accepted by physicians in public hospitals in Bangladesh, and its use may extend to that of other healthcare providers with less formal training.

## Supplemental files

10.4269/ajtmh.23-0444 Supplemental Materials
