# Supplementary material for: Qualitative Assessment of a Smartphone-Based Mobile Health Tool to Guide Diarrhea Management in Bangladesh
Source: Am J Trop Med Hyg. 2023 Dec 11;110(1):159–64. doi: 10.4269/ajtmh.23-0444 (PMC10793020; doi:10.4269/ajtmh.23-0444)
Supplement: Supplemental Materials [file tpmd230444.SD1.pdf]

Supplementary Appendix 1:

Overview of the qualitative interview guide

Guideline for informal interview with admitting physician(s)

Introduction

I would appreciate if you kindly allow me to ask some details about you and your job for the purpose of this important research work. Your responses are very important for this important work.

- In which position you are currently holding and how long?
- Highest educational grade attained? Years of graduation?
- Are you originally from this area/district?
- Your age in years.
- How long you have been working in this hospital?
- What are your major responsibilities at this hospital?
- What types of health services are available in this hospital?

Now I am going to ask you some questions about your experiences as a provider in this hospital.

- In your single shift how many diarrheal patients did you see in the Outpatient Department (OPD)/diarrheal ward? Can you explain detail about your working hour and workload?
- How much time you spend per patient in an OPD/diarrheal ward? Do you think this time is sufficient? Why or why not?
- In your opinion, what do patients (adults and children) expect from consultations when they attend here with diarrhea?

Questions related to the intervention (Applicable only after intervention)

- What is your experience regarding the smartphone-based decision support tool/ DEP?
- What are the advantages of these tools according to you? What are the challenges, if any?
- Are you aware of any weaknesses in your current strategy or decision support tool for prescribing IVs, antibiotic, zinc? Probes: What are the strengths and limitations of these?
- What barriers you faced to use this tool? Probes: What problems could you foresee and what ways do you think these might be solved?
- What do you think about the research staff involved in the project? Do they like being part of a research project? Why or why not?
- What behavioral changes you observed after using this Smartphone based decision support tool/DEP?
- What suggestions can you give for how to promote the use of smartphone-based decision support tools/DEP amongst health care providers like you.

Closing

- Is there anything else you think is important related to decision support tools or our intervention that we have not talked about?
- What other interventions, initiatives or policies are currently being implemented to improve diarrheal disease management at these hospitals?
